# Trends in health-related behaviors of Korean adults: study based on data from the 2008-2014 Community Health Surveys

**DOI:** 10.4178/epih/e2015042

**Published:** 2015-09-29

**Authors:** Yang Wha Kang, Yun Sil Ko, Keon Yeop Kim, Changhyun Sung, Dong Han Lee, Eunkyeong Jeong

**Affiliations:** 1 Division of Chronic Disease Control, Center for Disease Prevention, Korea Centers for Disease Control and Prevention, Cheongju, Korea; 2 Department of Preventive Medicine, Kyungpook National University School of Medicine, Daegu, Korea; 3 Division of Health Promotion, Ministry of Health and Welfare, Sejong, Korea

**Keywords:** Community Health Survey, Health-related behavior, Korea, Trend

## Abstract

Unhealthy lifestyle behaviors such as smoking, alcohol consumption, and physical inactivity (multiple risks) often lead to serious health consequence and impaired health status. The purpose of this study was to investigate the trend in health-related behavioral factors over time among adults in South Korea (hereafter Korea). The data of 1,595,842 Koreans older than 19 years who participated in the 2008-2014 Korea Community Health Survey were analyzed to assess the trend in the prevalence of behavioral risk factors. Individual or clustering health-related behaviors were assessed according to sex, age, and region among 228,712 adults who participated in the 2014 survey. From 2008 to 2014, the age-adjusted prevalence of obesity and high-risk alcohol use increased the prevalence of male current smoking and marginally decreased walking ability. Over 7 years, the percentage of adults who reported having all three healthy behaviors (i.e., currently not smoking, not consuming alcohol or having high-risk alcohol use, and engaging in walking) decreased from 35.2% in 2008 to 29.6% in 2014. Increased efforts to emphasize multiple health-related behavioral risk factors, including reducing alcohol use and smoking, and to encourage walking are needed in the thirties and forties age groups in Korea.

## INTRODUCTION

As South Korea (hereafter Korea) continues to develop economically, the average life expectancyof its population has also increased from 61.9 years in 1970 to 81.4 years in 2012. However, the current disability adjusted life expectancy is 73.0 years, showing a gap with the average life expectancy [1,2]. A disparity in community health status based on standardized mortality ratio was observed between regions [3]. Moreover, the number of patients with chronic illnesses is increasing owing to increasing population of elderly individuals older than 65 years. This has led to an increased interest in the burden of disease and health-related quality of life. The gap between the average life expectancy and disability-adjusted life expectancy, the health disparity between regions, and the trends in disease patterns can be considered as results of socioeconomic developments, changes in lifestyle, and regional differences. According to the Alameda County Study, not smoking, reduced alcohol consumption, regular physical activity, maintenance of a healthy weight, adequate amount of sleep, regular breakfast consumption, and snacking were proposed as health-related behavioral factors. Furthermore, these healthy behaviors correlated with mortality rate, either independently or in combination [4]. Healthy behaviors such as not smoking, moderate alcohol consumption, exercise, and healthy eating habits are known to prevent chronic diseases, including cancer, stroke, and cardiovascular diseases. Hence, we used the Korean Community Health Survey (CHS) to survey adults older than 19 years in Korea in order to investigate changes in main health-risk behaviors (smoking, alcohol consumption, engaging in walking, and obesity) and multiple health-related behaviors, as well as regional differences in these changes, over a period of 7 years (2008 to 2014).

## MATERIALS AND METHODS

We analyzed long-term data collected through the CHS over the period 2008 to 2014. The survey population of the CHS consisted of adults older than 19 years who were living residential housing (apartments and regular houses) in tong, ban/li, areas, in 254 cities, counties, and districts throughout the country. In the CHS, sample points were determined by using tong, ban/li, as the primary sampling unit, and the households surveyed were determined by using household as the secondary sampling unit. The final study subjects consisted of approximately 230,000 individuals for each year and 254 health centers from across the country, with an average of 450 households or approximately 900 household members per health center. Each result was weighted accordingly to represent local residents and was normalized for sex and age based on the estimated population in 2005 [5].

Among the indexes calculated by using data from the CHS, smoking, alcohol consumption, walking, and obesity were used as variables in the present study. First, to investigate changes in the main health-risk behaviors in the period 2008 to 2014, we examined the changes in the medians values of 4 indexes for each city and county, namely male current smoking rate, high-risk drinking rate, walking rate, and obesity rate (self-recorded). We then classified the indexes according to their rating in a scale of 10 and presented the data visually by using the geographic information system. Male current smoking rate was defined as the fraction of men who were currently smoking and had smoked more than 5 packs (100 cigarettes) in their lifetime. High-risk drinking rate was defined as the fraction of individuals who had been drinking in the recent year and drinking one sitting, at least twice per week, 7 drinks for males and 5 drinks for females. Walking rate was defined as the fraction of individuals who walked for at least 30 minutes, 5 days per week. Obesity rate (self-recorded) was defined as the fraction of individuals who had a body mass index greater than 25 kg/m^2^, calculated by using their known height and weight.

We examined the change in the number of three types of health-risk behaviors (not currently smoking, low-risk alcohol use, and engaging in walking) from 2008 to 2014. By using data from the 2014 CHS, we investigated the difference in multiple healthy behaviors according to sex, age, and region. Current non-smokers were defined as subjects who were not currently smoking. Low-risk alcohol drinkers were all the subjects who were not considered high-risk alcohol drinkers.

The study protocol was approved by the institutional review board (IRB) of Korea Centers for Disease Control and Prevention (IRB no. 2014-08EXP-09-4C-A).

## RESULTS

### Change in the indexes of the major health-risk behaviors (smoking, alcohol consumption, engaging in walking, and obesity) for each year (2008-2014)

**Figure 1.**
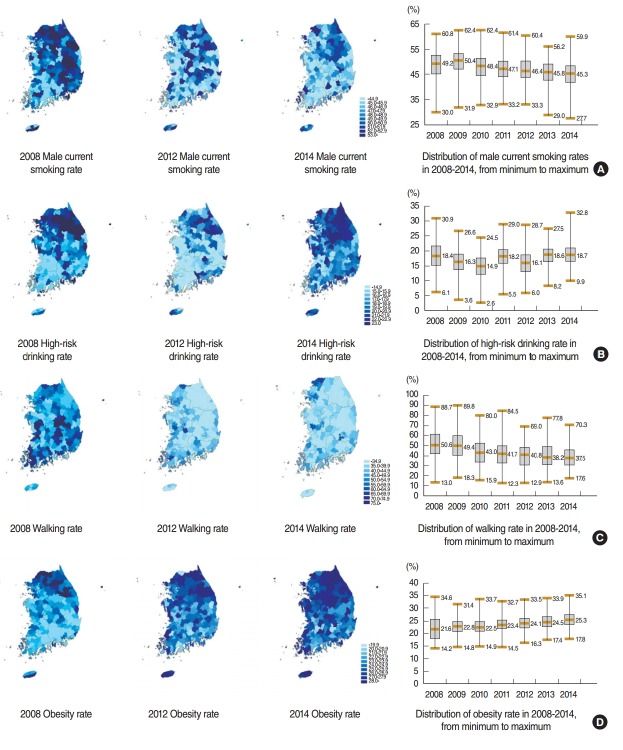
Age-adjusted and sex-adjusted trends in the prevalence of health-related risk behaviors during the period 2008-2014 in Korea, according to the Community Health Survey. (A) Male current smoking rate, (B) high-risk drinking rate, (C) walking rate, and (D) obesity rate.

The median male current smoking rate in each city, county, and district in the 7-year period increased marginally from 49.2% in 2008 to 50.4% in 2009. However, it has been continuously decreasing since then, from 48.4% in 2010 to 47.1% in 2011, 46.4% in 2012, 45.8% in 2013, and 45.3% in 2014. The regional difference in male current smoking rate (maximum value − minimum value) declined from 30.8% in 2008 to 27.1% in 2012 but increased to 32.2% in 2014. The high-risk drinking rate decreased from 18.4% in 2008 to 14.9% in 2010 and then increased back to 18.7% in 2014. The regional difference in high-risk drinking rate fluctuated from 24.8% in 2008 to 21.9% in 2010, 19.3% in 2013, and then to 22.9% in 2014. In particular, walking rate, which represents physical activity, continuously decreased from 50.6% in 2008 to 49.4% in 2009, 43.0% in 2010, 41.7% in 2011, 40.8% in 2012, 38.2% in 2013, and 37.5% in 2014. The regional difference in walking rate decreased from 75.7% in 2008 to 52.7% in 2014. Obesity rate (selfrecorded) increased from 21.6% in 2008to 22.8% in 2009, 22.5% in 2010, 23.4% in 2011, 23.4% in 2012, 24.1% in 2013, and 25.3% in 2014. The regional difference in obesity rate decreased from 20.4% in 2008 to 16.5% in 2013 but increased to 17.3% in 2014 (Figure 1).

### Practice rate for the main healthy behaviors(not smoking, low-risk alcohol use, and walking)

**Figure 2.**
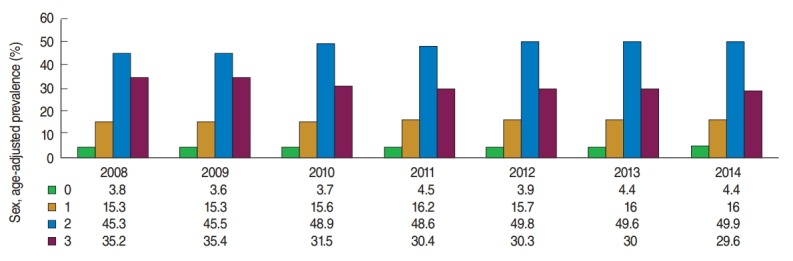
Sex-adjusted and age-adjusted prevalence of having 0 to 3 healthy behaviors, including not currently smoking, no alcohol consumption, no high-risk alcohol use, and engaging in walking as physical activity, during the period 2008-2014 in Korea, according to the Community Health Survey.

To understand the extent local residents practiced healthy behaviors over the 7-year period, we examined the change in the three main indexes they practiced. For the indexes investigated (not currently smoking, low-risk alcohol use, and walking), 3.7% to 4.5% of the subjects did not practice any of these behaviors. This percentage slightly increased from 2.8% in 2008 to 4.4% in 2013 and 2014. The percentage of those who practiced 1 or 2 of the behaviors showed an increasing trend every year from 60.6% in 2008 to 65.9% in 2014. Finally, the percentage of those who practiced all three indexes decreased in the past 7 years from 32.2% in 2008 to 29.6% in 2014 (Figure 2).

When examined according to age, the healthy behavior practice rates were lower in the thirties and forties age groups than in the other age groups. Of the subjects, 5.6% who were in their thirties and 6.6% who were in their forties did not practice healthy behaviors. These percentages were higher than the percentages in the twenties (3.1%), sixties (1.9%), and “older than 70 years” age groups (0.5%). All three healthy behaviors were practiced by 23.8% of the subjects in the thirties age group and by 25.0% of the subjects in the forties age group. These percentages were lower than that in the twenties (35.4%), sixties (37.2%), and “older than 70 years” age groups (33.5%). When differences were examined according to sex, females younger than 70 years (19 to 69 years old) were more prevalent than males of the same age range among those who practiced all three healthy behaviors. In particular, 40.9% of females in their fifties practiced all three healthy behaviors, indicating a 22.2% higher percentage than the percentage of males (18.7%). Moreover, the fifties age group showed most significant difference between males and females. By region, the prevalence of those who practiced all three healthy behaviors was highest in Seoul (39.3%), followed by Daejeon (34.5%) and Incheon (33.6%), and lowest in Jeju (21.3%), followed by Gyeongnam (21.5%) and Gyeongbuk (22.2%), showing regional differences. Furthermore, females were more likely to practice all three healthy behaviors in all regions, except Jeju. In particular, almost 20% more women than men living in Seoul, Busan, and Daegu practiced all three healthy behaviors, showing a large difference between sexes (Table 1).

## DISCUSSION

Based on the changes in the median values of the major health-risk behaviora lfactors according to city and county among adults in Korea, as shown in the CHS in the aforementioned 7-year period, the male current smoking rate is continuously declining. However, because of the constantly decreasing walking rate and the increasing obesity rate, an appropriate intervention is needed at both the national and local levels. Although regional differences in walking rate have been decreasing, the regional difference was as large as 52.7% (17.6% to 70.3%) in 2014. Regional differences in male current smoking and obesity rates had also been decreasing until recently but increased again to 32.3% and 17.3%, respectively, in 2014. Therefore, regional differences in male current smoking and obesity rates should be monitored.

In terms of the change in the practice rate of the three representative healthy behaviors (not smoking, reduced alcohol consumption, and walking) over the 7-year period, the number of individuals who practiced 1 or 2 of the healthy behaviors increased, whereas the number of those who practiced all three healthy behaviors decreased. Individuals who practiced all three healthy behaviors accounted for 29.6% of the subjects in the 2014 CHS, and those who did not practice any of the three healthy behaviors accounted for 4.4%. Because the practice rate of healthy behaviors was lower in males than in females and in those in their thirties and forties than in those in their twenties and fifties, local health promotion strategies that target these age groups are needed. The practice rate for healthy behaviors in 17 metropolitan areas showed regional differences. Therefore, measures to improve health-related behaviors should be implemented at the metropolitan level for regions with low practice rates.

Healthy behaviors influence the development of cardiovascular diseases, the incidence of cancer, general mortality and morbidity, and health status. In particular, smoking is the most important predictive factor of diseases and death, and has been highlighted as the priority of health objectives at the national level [6,7]. Excessive drinking can cause cerebrovascular diseases by increasing blood pressure [8], and obesity and lack of walking or other exercises affect morbidity and mortality, showing a close relationship to cardiovascular diseases [9-11]. In particular, for individuals who practice good healthy behaviors, the average life expectancy is 11 years longer than those who do not practice good healthy behaviors. Because 28% of males and 43% of females who ranked highly in practicing healthy behaviors showed low mortality rates, healthy behaviors were reported to affect mortality rate [12]. Ultimately, practicing healthy behaviors not only improves individual health status and prevents diseases but also contributes to health promotion among community residents. Along with the use of an individual approach that focuses on improving individual lifestyle for health promotion, a population approach that targets communities and improves local health behavioral factors should be developed in the future, both at the regional and national levels. To achieve this, CHSs must be continuously administered to provide systematic and accumulated community health data for research studies that investigate regional differences and changes in community health indexes. Studies on community health outcomes of community interventions and follow-up observations are also needed.

## Figures and Tables

**Table 1.** Trend of the regional prevalence of health-related behaviors in 2014, according to the Community Health Survey (n = 228,721)

	Health-related behaviors (%)											
	Total				Male				Female			
	0^1^	1	2	3	0	1	2	3	0	1	2	3
Overall	4.4	16.0	49.9	29.6	8.2	28.1	42.2	21.3	0.5	4.2	57.5	37.6
Age (yr)												
19-29	3.1	13.8	47.6	35.4	5.2	21.9	43.6	29.2	0.9	5.2	51.8	42.0
30-39	5.6	19.4	51.2	23.8	10.1	32.8	41.0	15.9	0.8	5.3	61.9	32.0
40-49	6.6	19.7	48.6	25.0	12.5	34.4	38.8	14.3	0.5	4.5	58.7	36.1
50-59	4.5	16.9	48.7	29.8	8.7	30.3	42.2	18.7	0.3	3.4	55.3	40.9
60-69	1.9	10.6	50.3	37.2	3.8	20.5	46.6	28.9	0.2	2.0	53.4	44.3
≥70	0.5	6.4	59.3	33.5	1.4	13.5	49.4	35.4	0.0	2.4	65.0	32.4
Region												
Seoul	2.9	12.8	44.9	39.2	5.3	22.6	43.2	28.7	0.5	3.3	46.6	49.4
Busan	4.6	16.1	48.8	30.5	8.8	28.4	41.1	21.6	0.4	4.0	56.3	39.2
Daegu	3.8	17.1	49.4	29.6	7.3	30.8	41.4	20.4	0.2	3.6	57.3	38.6
Incheon	4.0	16.1	46.2	33.6	7.2	27.2	41.5	24.0	0.9	5.2	50.8	43.0
Gwangju	4.6	16.2	55.2	24.0	8.8	29.1	44.9	17.3	0.5	3.6	65.3	30.6
Daejeon	3.4	14.9	47.0	34.5	6.2	25.7	41.9	25.9	0.7	4.3	51.9	42.9
Ulsan	4.0	15.9	52.5	27.6	7.5	28.1	42.6	21.9	0.6	4.0	62.2	33.1
Sejong	4.2	15.7	56.0	24.2	8.2	27.0	45.6	19.2	0.2	4.6	66.1	29.1
Kyonggi	4.7	16.4	50.9	28.0	8.9	28.6	42.5	19.9	0.6	4.4	59.0	35.9
Gangwon	6.0	19.2	52.4	22.3	11.6	32.0	39.9	16.5	0.6	6.5	64.7	28.1
Chungbuk	5.8	18.8	52.3	23.1	11.0	31.6	41.1	16.3	0.8	6.3	63.2	29.7
Chungnam	5.0	17.0	54.2	23.7	9.5	29.8	42.1	18.4	0.7	4.4	66.0	28.8
Jeonbuk	4.2	17.1	54.3	24.4	8.2	30.2	43.0	18.5	0.2	4.2	65.5	30.1
Jeonnam	4.1	15.8	50.9	29.1	8.0	28.6	41.7	21.5	0.2	3.3	59.9	36.4
Gyeongbuk	5.7	17.6	54.5	22.2	10.9	31.7	42.5	14.9	0.6	3.8	66.3	29.3
Gyeongnam	6.0	18.9	53.6	21.5	11.7	32.6	40.8	14.8	0.5	5.4	66.2	27.9
Jeju	5.9	19.1	53.6	21.3	10.7	33.4	39.3	16.6	1.2	5.0	67.6	26.0

1 Having 0 to 3 healthy behaviors, including not currently smoking, no alcohol consumption, no high-risk alcohol use, and engaging in walking as physical activity.
