# Supplementary material for: Trends in health-related behaviors of Korean adults: study based on data from the 2008-2014 Community Health Surveys
Source: Epidemiol Health. 2015 Sep 29;37:e2015042. doi: 10.4178/epih/e2015042 (PMC4835711; doi:10.4178/epih/e2015042)
Supplement: Supplementary file 1 [file epih-37-e2015042-supplementary.pdf]

Trends in health-related behaviors among adults in Korea

-Based on 2008～2014 Community Health Surveys-

Kang Yang[1], KoYun Sil[1], Kim Keon Yeop[2], Sung Changhyun[3], Kim Dong Han[1], Jeong Eunkyeong[1] ※

[1] Division of Chronic Disease Control, Korea Centers for Disease Control and Prevention

[2] Department of Prevention medicine, School of Medicine, Kyungpook National University

[3] Division of Health Promotion, Ministry of Health Welfare

※Corresponding author. Korea Centers for Disease Control and Prevention, Osong Health Technology Administration Complex.187 Osongsaengmyeong2(i)-ro, Cheongwon-gun, Chungbuk 363-951, E-mail:jeong68@korea.kr

Funding: This research was supported by a fund (090-091-4800-4849-306) from the Health Promotion Fund of Korea.

Running title: health-related behaviors among adults in Korea

Abstract

Background: Unhealthy lifestyle behaviors such as smoking, alcohol consumption, physical inactivity (multiple risks) often lead to serious consequence on the health and impaired health status. The purpose of this study was to investigate trend of health-related behavioral factors over time among Korea adults.

Methods: Data from 1,595,842(N) aged 19 over years who participated in the 2008–2014 Korea Community Health Survey were analyzed to assess the trend in the prevalence of behavioral risk factors. Having individual or clustering healthy behaviors by sex, age and region were also assessed among 228,712 adults in the 2014 survey.

Results: . From 2008 to 2014, the age-adjusted prevalence of obesity and high-risk alcohol use increased the prevalence of male current smoking and walking decreased marginally. Over the 7 years,

the percentages of adults reported having all three healthy behaviors (i.e., not currently smoking, no alcohol consumption or not high-risk alcohol use, engaging in walking) decreased from 35.2% in 2008 to 29.6% in 2014.

***Conclusions**:* Increased efforts emphasizing multiple health-related behavioral risk factors including reducing alcohol use and smoking and improving walking are needed that among under age 30-40 years old in Korea.

KEY WORDS: Community Health Survey, Health-related behavior, Korea, Trend

서론

우리나라는 경제발전과 더불어 평균수명이 1970년 61.9세에서 2012년 81.4세로 길어졌지만 2012년 현재 건강수명은 73.0세로 평균수명과는 차이(gap)가 있으며[1, 2], 표준사망비를 활용한 지역별 건강수준에서 지역간 건강불평등이 있음이 밝혀졌다[3]. 또한 65세 이상 노인인구가 지속적으로 증가함에 따라 만성질환자가 증가하고 있으며, 이로 인한 질병부담 및 건강관련 삶의 질에 대한 관심 또한 증가하고 있다. 이러한 평균수명과 건강수명의 차이, 지역간 건강차이, 질병양상의 추세는 사회경제적 발달과 함께 생활습관의 변화 및 지역간 차이 등에 그 원인이 있다고 볼 수 있다. Alameda county 연구에 따르면 건강수준과 연관성이 있는 건강행태로 금연, 절주, 규칙적인 신체활동, 건강한 체중유지, 적당한 수면, 규칙적인 아침식사 및 간식 여부 등을 제시하였고, 건강행위들이 독립적으로 혹은 복합적으로 사망률과 연관성이 있음을 밝혔다[4]. 금연, 적당한 음주, 운동, 건강한 식습관 등 건강생활실천은 암, 뇌졸중, 심혈관질환 등을 포함하는 만성질환을 예방하는 것으로 알려져 있다. 이에 최근 7년간(2008~2014) 우리나라 19세 이상 성인을 대상으로 조사된 지역사회건강조사 자료를 활용하여 주요 건강행태(흡연, 음주, 걷기실천, 비만) 및 다중건강생활실천행태(Multiple healthy behaviors)의 변화 및 지역간 차이를 살펴보고자 하였다.

연구대상 및 방법

분석 자료는 지역사회건강조사 2008년부터 2014년까지의 원시자료를 이용하였다. 지역사회건강조사의 조사모집단은 전국 254개 시·군·구내 통·반/리의 주거용 주택(아파트, 일반주택)에 거주하는 만 19세 이상 성인이다. 지역사회건강조사는 통·반/리를 1차 추출단위로 하여 표본지점을 추출하고, 가구를 2차 추출단위로 하여 조사대상가구를 추출한다. 최종 조사대상자는 매년 약 23만명이며 이는 전국 254개 보건소별 평균 450개 가구, 약 900여명의 가구원에 해당한다. 각 결과는 지역주민을 대표할 수 있도록 가중치를 부여하였으며 2005년 추계인구를 기준인구로 하여 성·연령

별 표준화한 값으로 제시하였다[5].

지역사회건강조사에서 산출한 지표들 중 본 연구에 사용된 변수로는 흡연, 음주, 걷기, 비만 등이었다. 먼저 주요 건강행태의 2008년부터 2014년까지 연도별 변화를 살펴보기 위해 남자현재흡연율, 고위험음주율, 걷기실천율, 비만율(자기기입) 4가지 지표의 시군구 중앙값 추이를 보고 이들 지표를 10등급으로 분류하여 GIS 도식화하였다. 남자현재흡연율은 평생 5갑(100개비) 이상 흡연한 사람으로서 현재 흡연하는 남자의 분율, 고위험음주율은 최근 1년 동안 음주한 사람 중에서 남자는 한 번의 술자리에서 7잔 이상, 여자는 5잔 이상을 주 2회 이상 마신다고 응답한 사람의 분율, 걷기실천율은 최근 1주일 동안 1회 30분 이상 걷기를 주 5일 이상 실천한 사람의 분율, 비만율(자기기입)은 본인이 알고 있는 키와 몸무게를 이용하여 계산한 체질량지수(Body, Mass Index, kg/㎡)가 25이상인 사람의 분율로 하였다.

건강행태 중 3가지 건강생활실천행태(현재금연, 저위험음주, 걷기실천)를 실천하는 개수의 변화를 2008년부터 2014년까지 살펴보았다. 2014년 지역사회건강조사에서 성, 연령, 지역별 다중건강생활실천행태의 차이를 살펴보았다. 현재금연은 현재흡연자를 제외한 모든 대상자, 저위험음주는 고위험음주에 해당되지 않는 모든 대상자가 해당된다.

연구결과

1. 주요 건강행태(흡연, 음주, 걷기실천, 비만) 지표의 연도별 변화(2008-2014년)

지난 7년간 남자현재흡연율의 시군구 중앙값은 2008년 49.2%에서 2009년 50.4%로 소폭 상승하였으나, 2010년 48.4%, 2011년 47.1%, 2012년 46.4%, 2013년 45.8%, 2014년 45.3%로 지속적으로 감소하고 있다. 남자현재흡연율의 지역간 차이(최대값-최소값)는 2008년 30.8% 포인트에서 2012년까지 27.1% 포인트로 지속적으로 감소하였지만 2014년에는 32.2% 포인트로 증가하였다. 고위험음주율의 경우 2008년 18.4%, 2010년 14.9%, 2014년 18.7%로 증감을 반복하였다. 고위험음주율의 지역간 차이는 2008년 24.8% 포인트, 2010년 21.9% 포인트, 2013년 19.3% 포인트, 2014년 22.9% 포인트로 증감을 반복하였다. 특히 신체활동을 대표하는 걷기실천율의 경우 2008년 50.6% 이후 2009년 49.4%, 2010년 43.0%, 2011년 41.7%, 2012년 40.8%, 2013년 38.2%, 2014년 37.5%로 지속적으로 감소하고 있다. 걷기실천율의 지역간 차이는 2008년 75.7% 포인트에서 2014년 52.7% 포인트로 감소하였다. 비만율(자기기입)은 2008년 21.6%에서 2009년 22.8%, 2010년 22.5%, 2011년 23.4%, 2012년 24.1%, 2013년 24.5%, 2014년 25.3%로 지속적으로 증가하고 있다. 비만율의 지역간 차이는 2008년 20.4% 포인트에서 2013년 16.5% 포인트로 감소하다가 2011년 17.3% 포인트로 증가하였다(Figure 1).

Figure1

2. 주요 건강생활행태(금연, 절주, 걷기실천)의 실천율

지난 7년간 지역주민의 건강생활 실천 정도를 파악하기 위해 주요 3개 지표를 기준으로 그 추이를 살펴보았다. 현재금연, 저위험음주, 걷기실천 지표 중 한 가지도 실천하지 않는 대상자는 3.7~4.5%였으며 2008년 3.8%에서 2013, 2014년 4.4%로 약간 증가하였다. 1~2가지 건강생활실천자는 2008년 60.6%에서 2014년 65.9%로 매년 증가하는 추세이다. 3가지 모두 실천자는 29.6~35.4%였으며 2008년 35.2%에서 2014년 29.6%로 지난 7년 간 감소하는 추세였다(Figure 2).

Figure2

연령별로 살펴보면 30, 40대 젊은 층의 건강생활실천은 다른 연령층보다 좋지 않은 것으로 나타났다. 건강생활실천을 전혀 하지 않는 경우가 30대에서 5.6%, 40대에서 6.6%로 나타나 20대 3.1%, 60대 1.9%, 70대 이상 0.5%보다 높았고, 3가지 모두 실천하는 경우도 30대에서 23.8%, 40대에서 25.0%로 나타나 20대 35.4%, 60대 37.2%, 70대 이상 33.5%보다 낮았다. 성별에 따른 차이를 보면 70대 이하 모든 여성층(19~69세)에서 남성보다 3가지 모두 건강생활을 하는 경우가 높았으며, 특히 50대 여성의 경우 40.9%로 남성의 18.7%보다 22.2% 포인트가 높아 50대에서 남녀차이가 가장 컸다. 지역별로는 3가지 건강생활실천을 모두 실천한다는 응답자의 경우 서울 39.2%, 대전 34.5%, 인천 33.6%로 높았고, 제주 21.3%, 경남21.5%, 경북 22.2%로 낮게 나타나 지역 간 차이가 있었다. 또한 3가지 건강생활실천을 모두 실천한다는 응답자가 제주를 제외한 모든 지역에서 여성이 높았는데, 서울, 부산, 대구 여성의 경우 같은 지역 남성보다 최대 20% 포인트 이상 높아 큰 격차가 나타났다(Table 1).

Table1

결론

지난 7년간 지역사회건강조사결과에서 나타난 우리나라 성인들의 주요 건강행태 지표의 시군구 중앙값 추이를 보면 남자현재흡연율은 지속적으로 감소하고 있어 긍정적이었다. 하지만 걷기실천율은 지속적으로 감소하고 비만율은 지속적으로 증가하고 있어 국가 및 지역차원의 개입(intervention)이 필요하다. 걷기실천율의 경우 지역간 차이가 매년 감소하고는 있지만 2014년 52.7% 포인트(17.6%~70.3%)로 매우 컸으며, 남자현재흡연율과 비만율의 경우 지역간 차이가 최근까지 감소하다 2014년 각각 32.2% 포인트, 17.3% 포인트로 증가하여 향후 지역간 차이에 대한 추세를 지켜볼 필요가 있다.

금연, 절주, 걷기실천의 3가지 대표적인 건강생활실천율의 지난 7년간 추이를 보면, 1~2개 실천자수는 증가하고 있는데 반해 3개 실천자수는 감소하고 있다. 2014년 지역사회건강조사 대상자 중 3개 실천자는 29.6%를 차지하였고 한 개도 실천하지 않은 대상자가 4.4%였다. 여자보다는 남자에서, 20대 및 50대 이상보다는 30, 40대에서 건강생활실천율이 낮아 지역사회에서 이들을 대상(target)으로 한 건강증진사업 전략이 마련되어야 할 것이다. 17개 광역 단위로 본 건강생활실천율에서 지역간 차이가 나타나 실천율이 낮은 지역의 경우 광역단위의 대책 마련이 필요하다.

건강생활실천은 심혈관계질환, 암질환 발생률, 일반사망률과 이환율 및 건강수준에 미치는 요인으로, 흡연은 질병과 사망의 가장 중요한 결정요인으로서 국가적 차원의 보건목표 중에서도 우선순위가 가장 높은 것으로 강조되고 있다[6, 7]. 음주는 혈압을 상승시켜서 뇌혈관질환을 일으키며[8], 걷기 등 운동부족과 비만은 이환율과 사망률에 영향을 주는 데 특히 심혈관계 질환과 밀접한 연관이 있다[9~11]. 특히 좋은 건강행태실천을 하는 사람의 경우 평균수명이 그렇지 못한 사람에 비해 11년 이상 길고, 건강실천행위 상위군에 속하는 남자 28%, 여자 43%는 낮은 사망률을 보여 건강생활실천행위는 사망률에 영향을 주는 것으로 보고되고 있다[12]. 결국 건강생활실천은 개인의 건강 상태를 개선하고 질병을 예방할 수 있을 뿐만 아니라 지역사회 주민의 건강증진에 기여한다. 지역사회 및 국가에서는 개인을 대상으로 한 생활습관 개선이 중심이 되는 개인접근(individual approach) 건강증진사업과 함께 지역사회를 대상으로 한 지역수준의 건강행태지표를 개선하고자 하는 인구집단접근(population approach)의 건강증진사업이 향후 추진되어야 할 것이다. 이를 위해서는 지속적인 지역사회건강조사를 수행하여 체계적이고 누적된 지역건강자료를 활용한 지역 건강지표의 추이 및 지역간 차이 등을 밝혀내는 연구와 함께 지역개입 연구, 추적관찰을 통한 지역건강결과 연구 등이 필요하다.

참고문헌

1. Statistics Korea. Cause of Death Statistics 2013[cited 2015 May10]. Available from http://kosis.kr/statHtml/statHtml.do?orgId=101&tblId=DT_1B34E13&conn_path=I3(Korea).

2. Korea Health Promotion Foundation. Health Plan 2020 Annual Report 2014[cited 2015 May10]. Available from http://www.khealth.or.kr/BoardType67.do?bid=62&mid=361&cmd=_view&dept=&mnu=&smnu=&idx=8509(Korea).

3. Kim JH, Yoon TH. Comparisons of Health Inequalities in Small Areas with Using the Standardized Mortality Ratios in Korea. Journal of preventive medicine and public health 2008;41:300-306.

4. Housman J, Dorman S. The Alameda County Study: A Systematic, Chronological Review American Journal of Health Education 2005;36:302-308.

5. Korea Centers for Disease Control and Prevention. Community Health Survey [cited 2015 Aug 12]. Available from: https://chs.cdc.go.kr/chs/ (Korean).

6. US Department of Health and Human Service. The Health Consequences of Smoking-50 Years of Progress. A Report of the Surgeon General Executive Summary: Public Health Service Office of the Surgeon General. 2014.

7. Kang HY, Kim HJ, Park TK, Nam CM, Park HW. Economic burden of smoking in Korea. Tobacco Control. 2003;12:37-44.

8. Rantakomi SH. The frequency of alcohol consumption is associated with the stroke mortality. Acta Neurologica Scandinavica 2014;130:118-124.

9. Kokkinos P. Cardiorespiratory fitness, exercise, and blood pressure. Hypertension 2014;64:1160-1164.

10. Mathieu RA, Powell-Wiley TM, Ayers CR, McGuire DK, Khera A, Das SR, et al. Physical Activity Participation, Health Perceptions, and CVD Mortality in a Multi-Ethnic Population: The Dallas Heart Study. Am Heart J 2012;163:1037–1040.

11. Froelicher VE. The effects of chronic exercise on the heart and on coronary atherosclerotic heart disease; A literature survey. Report SAM-TR 76-6 to the USAF School of Aerospace Medicine.1976.

12. Nandi AI, Glymour MM, Subramanian SV. Association among socioeconomic status, health behaviors, and all-cause mortality in the United States. Epidemiology 2014;25:170-177.

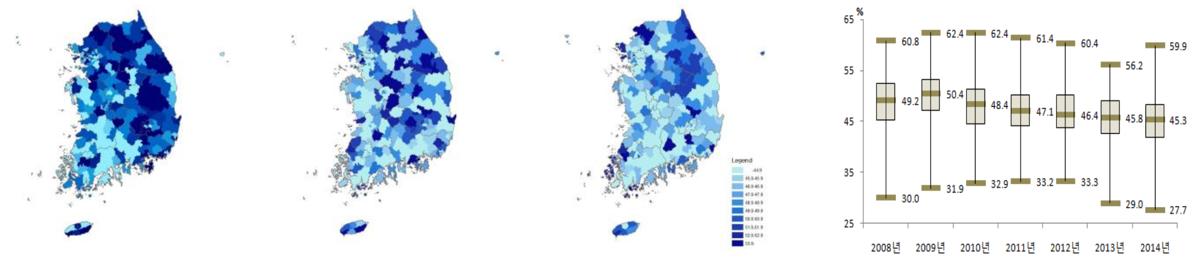

2008 Male current smoking Rate

2012 Male current smoking Rate

2014 Male current smoking Rate

Distribution for current smoking rate of male in 2008-2014 ranging from a minimum to a maximum

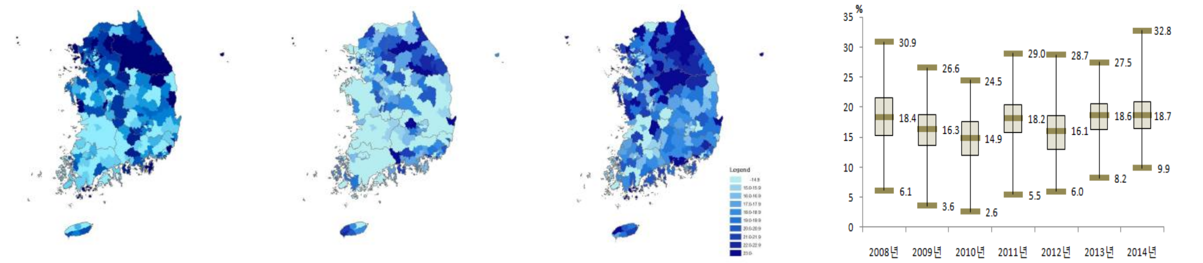

2008 High-Risk Drinking Rate

2012 High-Risk Drinking Rate

2014 High-Risk Drinking Rate

Distribution of High-Risk Drinking Rate in 2008-2014 ranging from a minimum to a maximum

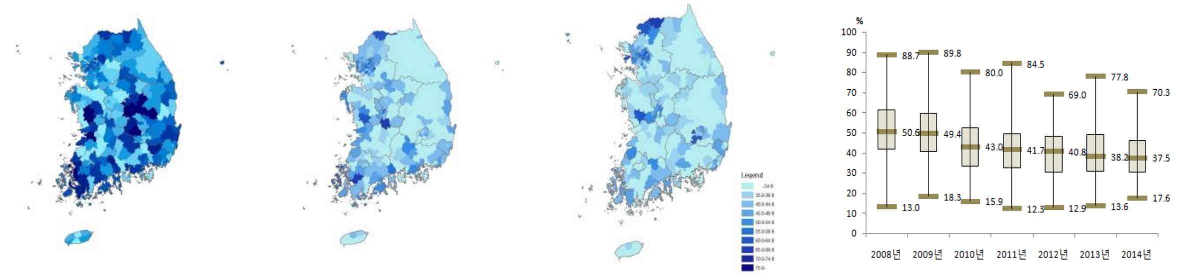

2008 Walking Rate

2012 Walking Rate

2014 Walking Rate

Distribution for Walking Rate in 2008-2014 ranging from a minimum to a maximum

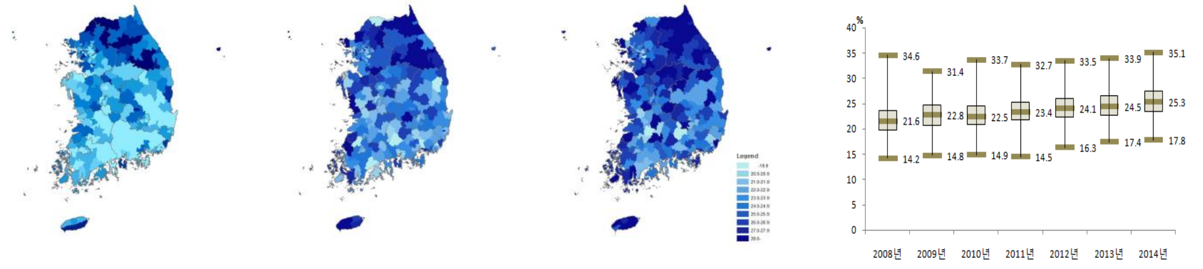

2008 Obesity Rate

2012 Obesity Rate

2014 Obesity Rate

Distribution for Obesity Rate in 2008-2014 ranging from a minimum to a maximum

Figure 1. Age, Sex-adjusted trends in prevalence of Health-related risk behaviors during the period of 2008-2014 in Korea, Community Health Survey.

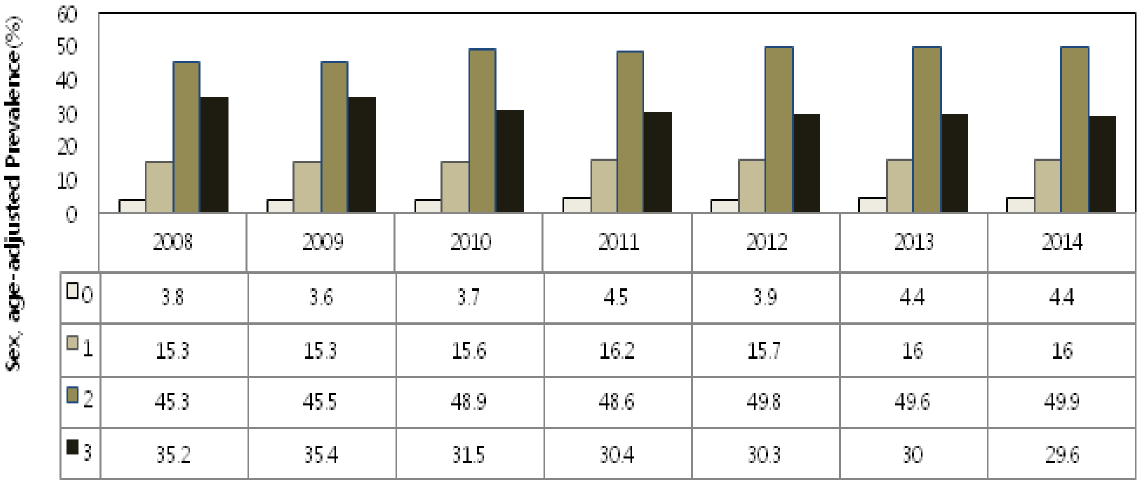

	2008	2009	2010	2011	2012	2013	2014
0	3.8	3.6	3.7	4.5	3.9	4.4	4.4
1	15.3	15.3	15.6	16.2	15.7	16	16
2	45.3	45.5	48.9	48.6	49.8	49.6	49.9
3	35.2	35.4	31.5	30.4	30.3	30	29.6

Figure 2. Sex, Age-adjusted prevalence of having 0 to 3 healthy behaviors including not currently smoking, no alcohol consumption or not high-risk alcohol use, engaging in walking physical activity during the period of 2008–2014 in the Korea, Community Health Survey.

Table1. Trend of regional in prevalence of Health-Related Behaviors during the period of Community Health Survey 2014 (N= 228,721)

	Health-Related Behaviors (%)											
	Total				Male				Female			
	0[1]	1	2	3	0	1	2	3	0	1	2	3
Overall	4.4	16.0	49.9	29.6	8.2	28.1	42.2	21.3	0.5	4.2	57.5	37.6
Age												
19-29	3.1	13.8	47.6	35.4	5.2	21.9	43.6	29.2	0.9	5.2	51.8	42.0
30-39	5.6	19.4	51.2	23.8	10.1	32.8	41.0	15.9	0.8	5.3	61.9	32.0
40-49	6.6	19.7	48.6	25.0	12.5	34.4	38.8	14.3	0.5	4.5	58.7	36.1
50-59	4.5	16.9	48.7	29.8	8.7	30.3	42.2	18.7	0.3	3.4	55.3	40.9
60-69	1.9	10.6	50.3	37.2	3.8	20.5	46.6	28.9	0.2	2.0	53.4	44.3
70 over	0.5	6.4	59.3	33.5	1.4	13.5	49.4	35.4	0.0	2.4	65.0	32.4
Region												
Seoul	2.9	12.8	44.9	39.2	5.3	22.6	43.2	28.7	0.5	3.3	46.6	49.4
Busan	4.6	16.1	48.8	30.5	8.8	28.4	41.1	21.6	0.4	4.0	56.3	39.2
Daegu	3.8	17.1	49.4	29.6	7.3	30.8	41.4	20.4	0.2	3.6	57.3	38.6
Incheon	4.0	16.1	46.2	33.6	7.2	27.2	41.5	24.0	0.9	5.2	50.8	43.0
Gwangju	4.6	16.2	55.2	24.0	8.8	29.1	44.9	17.3	0.5	3.6	65.3	30.6
Daejeon	3.4	14.9	47.0	34.5	6.2	25.7	41.9	25.9	0.7	4.3	51.9	42.9
Ulsan	4.0	15.9	52.5	27.6	7.5	28.1	42.6	21.9	0.6	4.0	62.2	33.1
Sejong	4.2	15.7	56.0	24.2	8.2	27.0	45.6	19.2	0.2	4.6	66.1	29.1
Kyonggi	4.7	16.4	50.9	28.0	8.9	28.6	42.5	19.9	0.6	4.4	59.0	35.9
Gangwon	6.0	19.2	52.4	22.3	11.6	32.0	39.9	16.5	0.6	6.5	64.7	28.1
Chungbuk	5.8	18.8	52.3	23.1	11.0	31.6	41.1	16.3	0.8	6.3	63.2	29.7
Chungnam	5.0	17.0	54.2	23.7	9.5	29.8	42.1	18.4	0.7	4.4	66.0	28.8
Jeonbuk	4.2	17.1	54.3	24.4	8.2	30.2	43.0	18.5	0.2	4.2	65.5	30.1
Jeonnam	4.1	15.8	50.9	29.1	8.0	28.6	41.7	21.5	0.2	3.3	59.9	36.4
Gyeongbuk	5.7	17.6	54.5	22.2	10.9	31.7	42.5	14.9	0.6	3.8	66.3	29.3
Gyeongnam	6.0	18.9	53.6	21.5	11.7	32.6	40.8	14.8	0.5	5.4	66.2	27.9
Jeju	5.9	19.1	53.6	21.3	10.7	33.4	39.3	16.6	1.2	5.0	67.6	26.0

[1] Having 0 to 3 healthy behaviors including not currently smoking, no alcohol consumption or not high-risk alcohol use, engaging in walking physical activity
